# A Low-Cost Dual-Frequency Dual-Polarized Antenna Array with High Gain

**DOI:** 10.3390/mi16101183

**Published:** 2025-10-19

**Authors:** Jin-Dong Zhang, Min Wang, Wen Wu

**Affiliations:** Key Laboratory of Near-Range RF Sensing ICs and Microsystems (NJUST), Ministry of Education, School of Electronic and Optical Engineering, Nanjing University of Science and Technology, Nanjing 210094, China; wangmin@mail.njust.edu.cn (M.W.); wuwen@njust.edu.cn (W.W.)

**Keywords:** dual frequency, dual polarization, high gain, low cost, radial power divider

## Abstract

A high-gain microstrip antenna array is proposed. The dual-frequency and dual-polarization characteristics of the array allow a satellite communication system to transmit and receive signals with a single antenna. To avoid high losses in microstrip feed lines for large apertures, the array is divided into subarrays, each fed by a low-loss separate feed network. The dual-frequency dual-polarization function is realized by utilizing two orthogonal modes of a corner-fed rectangular patch in a single-layer substrate. Moreover, to minimize losses in the separate feed network, semi-ridged coaxial lines and five four-way radial power dividers are employed. The power divider, composed of a cylindrical cavity and five SMA connectors, features very low insertion loss. Finally, to validate the design concept, a prototype of the proposed 32 × 32-element array operating at 12.5 GHz and 14.25 GHz is fabricated and measured. The measured results are in good agreement with the simulated ones. The −10 dB return loss frequency bands for the two operating frequencies are 12.04 GHz–12.69 GHz and 13.82 GHz–14.66 GHz, respectively. The measured gains at the two operating bands are 34.5 dBi and 35.2 dBi, respectively.

## 1. Introduction

Recently, due to the demand for satellite communications, the need for high-gain antennas is increasing [1]. Traditionally, reflector antennas are mostly used due to their high aperture efficiency; however, they are bulky and unsuitable for some applications. Planar antennas, which can be integrated with vehicles, are more desirable [2]. In addition to high gain, there are requirements for frequency and polarization. The uplink and downlink of most satellites operate at two different frequencies and polarizations. Usually, these two functions are realized by two separate antennas. The size can be significantly reduced if a single antenna with dual-frequency dual-polarization characteristics is used.

Several planar antennas have been reported to achieve high gain and dual-frequency dual-polarization. Waveguide slot arrays are good candidates for high gain and have been applied in many communication and radar systems [3,4]. In [5], dual-frequency dual-polarization is achieved using a parallel ridged waveguide. However, this antenna employs a complex multilayer feed network, which makes the entire antenna complex and difficult to fabricate. To avoid a multi-layer structure, a post-wall waveguide slot array with an interdigital structure is proposed in [6]. Two slotted arrays operating at dual polarizations are arranged on the same plane. However, to reduce the physical dimension of the waveguide width and resultant grating lobes, the waveguides must be filled with dielectric, resulting in low efficiency.

Radial-line slot-array antennas (RLSAs) are another type of low-profile antennas capable of high gain for microwave and millimeter-wave bands [7]. Various RLSAs have been applied in direct broadcast from satellite (DBS) systems. The problem with RLSAs is their limited design flexibility. The rigidity of the feeding network makes it difficult to achieve dual-frequency dual-polarization features. Although dual-polarization RLSAs have been developed, they operate at a single frequency [8,9].

Microstrip patch antennas are popular for their low profile and light weight. In contrast to waveguide slot arrays or RLSAs, they can easily realize dual-frequency dual-polarization [10,11,12,13,14,15,16,17,18,19]. The main disadvantage of microstrip arrays is efficiency limitations for large arrays, where the feed network is large and complex, resulting in high losses from feed lines [20].

In this paper, a low-cost, high-gain dual-frequency dual-polarization microstrip array is proposed. Specifically, the detailed design of a 32 × 32-element array operating at 12.5 GHz and 14.25 GHz is presented. The large array is divided into smaller subarrays. To avoid high losses in microstrip feed lines, these subarrays are fed by a low-loss separate feed network consisting of semi-ridged coaxial lines and low-loss power dividers. This array is low-cost, lightweight, and easy to fabricate.

## 2. Antenna Configuration

**Figure 1:**
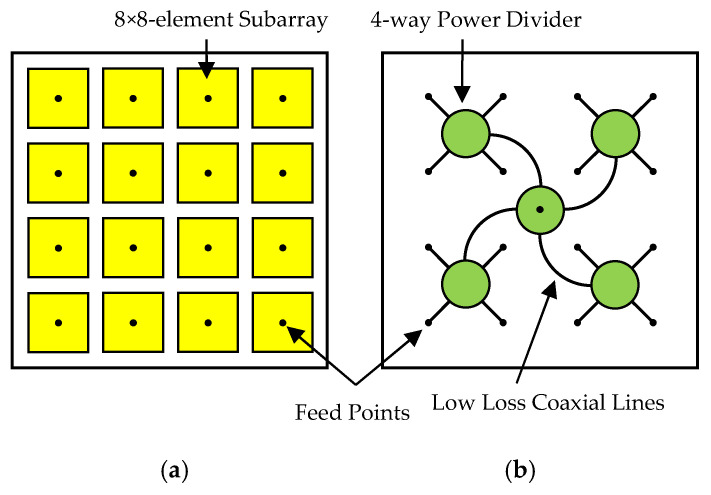
Configuration of the low-cost high-gain array. (**a**) Top view. (**b**) Bottom view.

To achieve high gain using a microstrip antenna array and avoid high insertion loss from microstrip feed lines, large arrays can be divided into subarrays. Each subarray is designed for high aperture efficiency, and all subarrays are fed by a low-loss feed network. For example, the 32 × 32-element array (Figure 1) is divided into sixteen 8 × 8-element subarrays. Each subarray is fed by a low-loss semi-ridged coaxial line, and all these semi-ridged coaxial lines are combined into a single output port through five four-way power dividers. In addition to low loss, this configuration offers portability and flexibility: it can be separated into parts for transport and easily reassembled into different sizes.

## 3. Antenna Design

### 3.1. Low-Loss Feed Network

The separate low-loss feed network (Figure 1b) consists of five four-way power dividers and twenty semi-ridged coaxial lines (commercially available with low loss). The power divider is designed to achieve extremely low insertion loss, balanced amplitude and phase outputs, high isolation, high power capacity, and dual-band coverage.

Among existing power dividers, radial dividers are promising due to their axially symmetric structure, which ensures good amplitude and phase balance [21,22]. Unlike microstrip power dividers, their fields are mainly concentrated in air, reducing dielectric loss. Traditional radial dividers have narrow bandwidth, but this issue was resolved using a resonant-type cylindrical power divider [23]. Inspired by this, a four-way radial divider was designed and fabricated.

**Figure 2:**
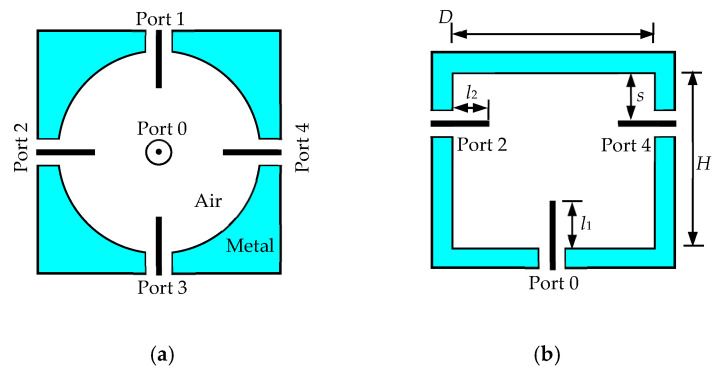
Geometry of the four-way radial power divider. (**a**) Top view (**b**) Front view.

The four-way radial power divider (Figure 2) consists of a cylindrical cavity and five SMA coaxial ports. One SMA connector is centered at the cavity bottom, with its inner conductor acting as a coupling probe. The other four SMA inner conductors are inserted symmetrically into the cavity. Proper adjustment of port positions and probe lengths ensures strong coupling between the input and output ports.

According to [24], the cavity diameter *D* should satisfy the following equation:

(1) $$\frac{5.52}{\pi f_{\max}\sqrt{\mu_{0}\varepsilon_{0}}}>D>\frac{2.405}{\pi f_{\min}\sqrt{\mu_{0}\varepsilon_{0}}}$$

where *ε*_0_ and *μ*_0_ are the permittivity and permeability of free space, respectively. The working frequency in this design is 12.5 GHz for receiving and 14.25 GHz for transmitting, each with 500 MHz operating band. To cover the above operating band, the range of *D* should between 18.7 mm and 36.3 mm according to (1). For this reason, the *D* is chosen to be 23.4 mm. According to the principles presented in [23], the initial values of other parameters can be obtained as detailed in Table 1. In this table, *λ_g_* is the wavelength of TM_011_ mode in cylindrical cavity which equals to 2.62 *D*, and *λ*_0_ is the wavelength of central frequency in free space which can be determined by

(2) $$\lambda_{0}=\frac{2c}{f_{\max}+f_{\min}}$$

where *c* is the speed of light in free space.

Once the initial values are obtained, a commercial simulation tool, CST-Microwave Studio (CST-MWS) 2021 is used to optimize the performance of the power divider. The optimized values are given in Table 1. Design Parameters for the Power Divider. As can be seen, the frequency bands where |S_00_| is below −10 dB are from 11.68 GHz to 15.2 GHz, thus fully covering the operating band. The simulated insertion loss for each port is less than 0.1 dB, which is much lower than that in microstrip power dividers.

**Figure 3:**
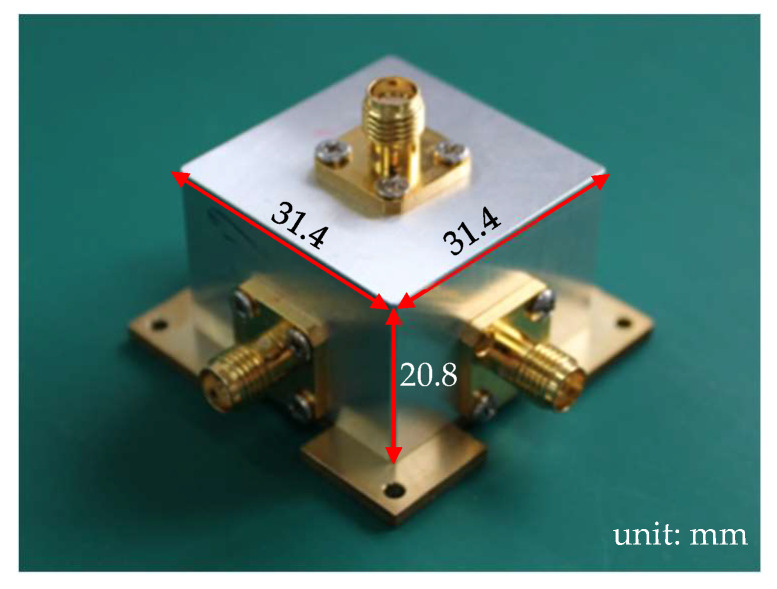
A photograph of the fabricated four-way radial power divider.

**Figure 4:**
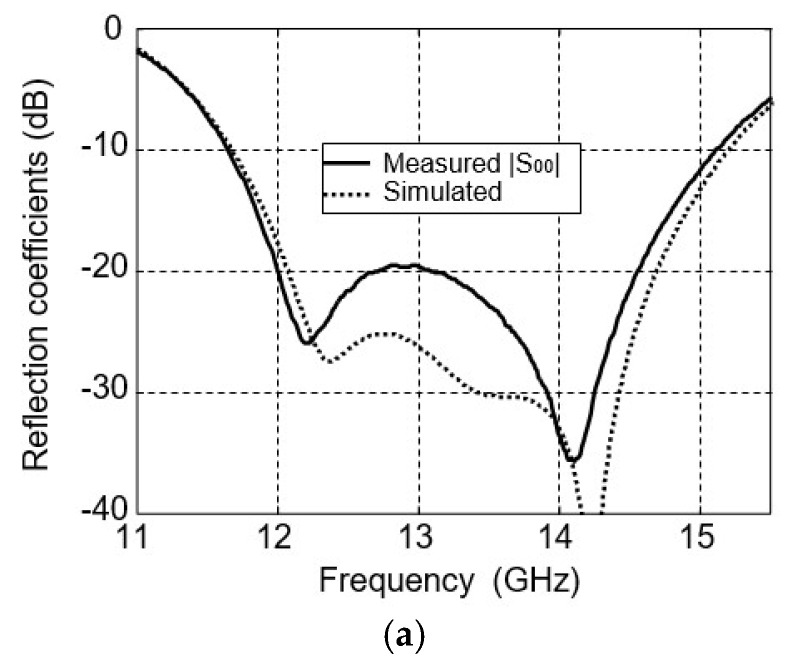

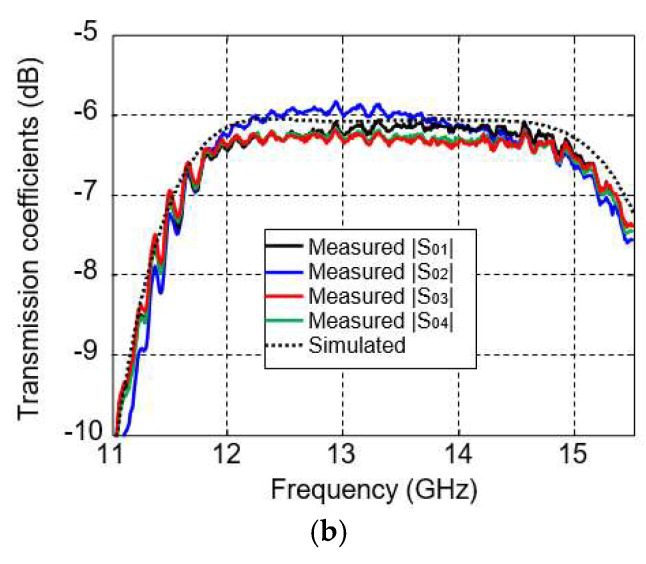
Simulated and measured results of power divider. (**a**) Reflection coefficients. (**b**) Transmission coefficients.

The radial power divider was fabricated from an aluminum block with dimensions of 31.4 × 31.4 × 20.8 mm^3^. Figure 3 shows the fabricated power divider. The thickness of the wall is 4 mm to fix the SMA connectors. This power divider has been measured by an Agilent network analyzer. For comparison, the measured results of S parameters are also shown in Figure 4, wherein the measured frequency bands that |S_00_| below −10 dB is from 11.65 GHz to 15.12 GHz. As expected, a good agreement is obtained between the simulated and measured frequency responses. The measured insertion losses of all the four ports in 12.5 GHz and 14.25 GHz are lower than 0.35 dB, which is a little higher than the simulated ones. Certain discrepancies exist in the measured data, ranging from 5.9 dB to 6.4 dB. This is mainly attributed to the inability to achieve complete uniformity among the SMA connectors we used, as well as slight differences in the insertion depth of the probes.

### 3.2. Dual-Frequency Dual-Polarization Subarray

**Figure 5:**
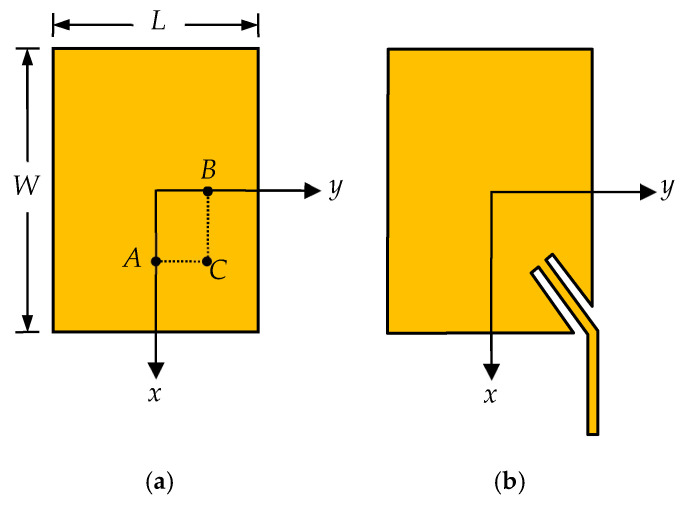
Geometry of the dual-frequency dual-polarization antenna element. (**a**) Basic principle. (**b**) The feed method used in this paper.

There are many papers published in the past to realize dual-frequency or even multi-frequency using microstrip patches, such as using reactively loaded or multilayer stacked structures [10,11]. Most of them are operating with single polarization. The dual-frequency patches operating with different polarizations mostly employ the orthogonal modes of a patch; Figure 5 shows an example of a rectangular patch, where matching can be achieved if feed point *A* is adjusted properly along *x*-axis and TM_01_ mode can be excited. Matching can also be achieved if the feed point *B* is adjusted along *y*-axis and TM_10_ mode can be excited. The resonant frequencies *f*_01_ and *f*_10_ mainly depend upon rectangular patch dimensions (*W* and *L*) and the substrate permittivity.

There are two feed methods to achieve the dual-frequency and dual-polarization characteristic simultaneously. One is using two separate feed lines as is done in [12], each feed line is responsible for one frequency and one polarization. The feed lines and the radiating patch are arranged in two different layers in this structure, making the antenna complicated and costly. In addition, the arrangement of the feed lines will become a big challenge if the element is used to form an array. Crossover between feed lines cannot be avoided and much insertion loss will be introduced. Therefore, the structure with single layer and single feed point is desirable. Such a structure has been reported in [13], where the patch is excited neither at point A nor point B, but at point C. The element using this method is not only compact in size and low cost but also easier to design. However, the element is fed by a coaxial probe, and not suitable to form a large array. To address this issue, a corner-fed method was proposed previously [18], as shown in Figure 5b. A microstrip line is inserted into one edge to achieve the simultaneous matching at the two frequencies. The influence of the insert angle and the detail design method are also researched.

**Figure 6:**
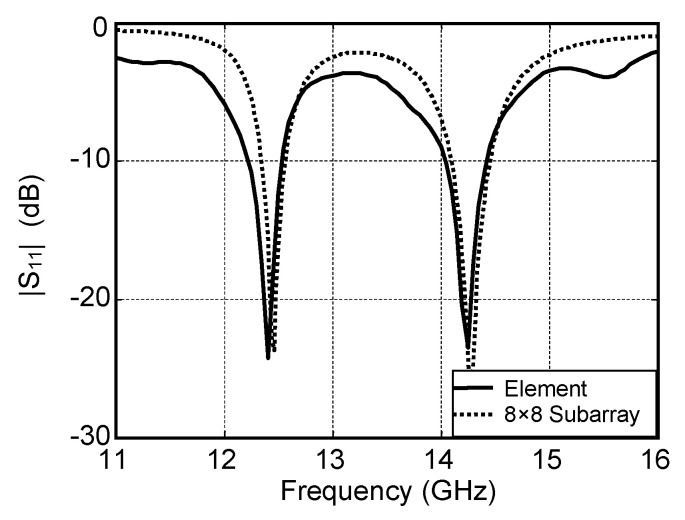
Simulated S-parameters of element and 8 × 8-element subarray.

In this paper, the single element proposed in [18] operating at 12.5 GHz and 14.25 GHz is used. The element is designed on an Arlon DiClad 880 (Chandler, AZ, USA) substrate with a thickness of 0.508 mm and ε_r_ = 2.2. The dimensions of the patch are fixed to be 7.46 mm × 6.54 mm. The element is simulated by another full wave simulation software HFSS V15. The simulated S parameter is shown in Figure 6. As is shown, the bandwidths for the two operating frequencies 12.5 GHz and 14.25 GHz are 1.7% and 2.1%, respectively.

**Figure 7:**
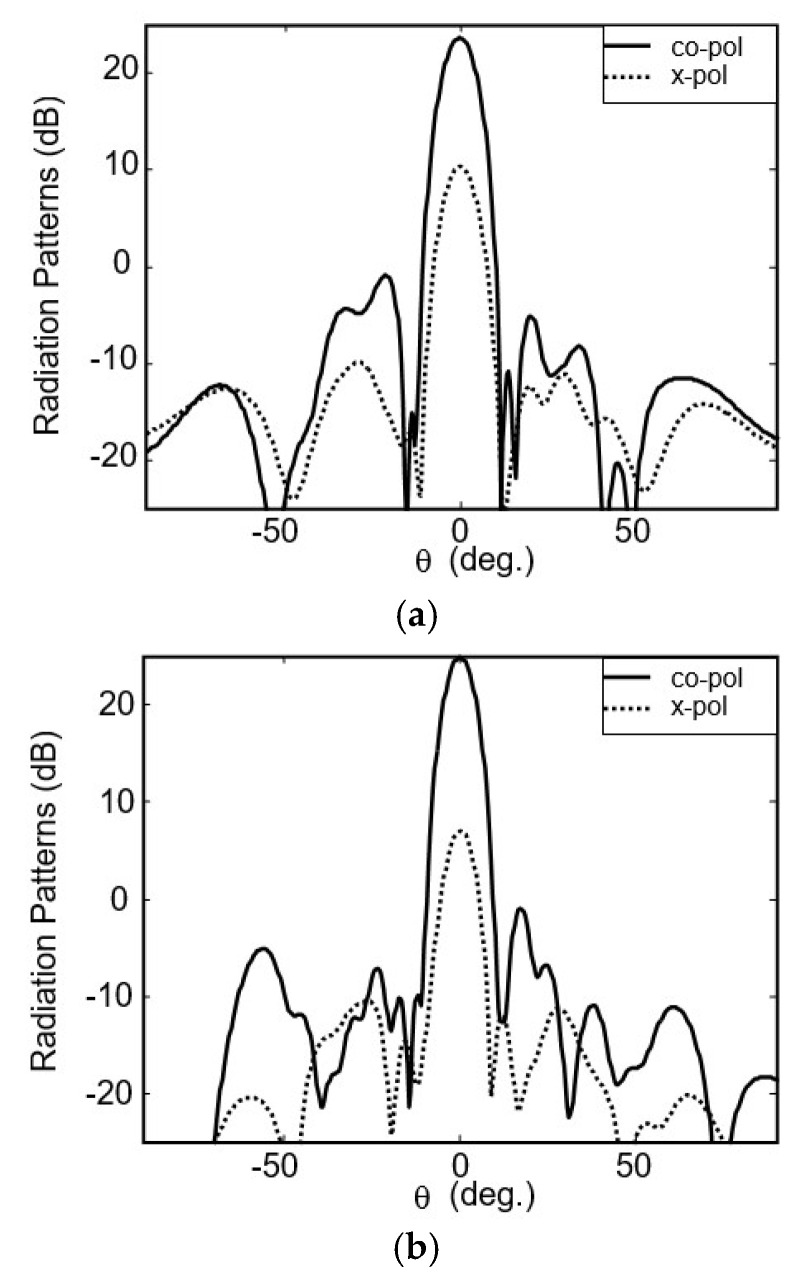
Simulated radiation patterns of 8 × 8-element subarray. (**a**) 12.5 GHz. (**b**) 14.25 GHz.

The element is chosen to be the basic building block to form a dual-frequency dual-polarization subarray. To achieve a high-gain low-cost array, the element number of subarray is an important parameter. The whole 32 × 32-element array can be composed of subarrays with 16 × 16 elements, 8 × 8 elements or even minor elements. Larger subarrays will introduce more insertion loss in their microstrip feed network. However, the corresponding separate feed network will be more simple and introduce less loss. The total loss is the sum of the loss in subarray and that in separate feed networks. Table 2 shows the simulated loss budgets of the 32 × 32-element array operating at 12.5 GHz when different subarrays are used. In this table, the insertion loss of each power divider and semi-ridged lines is fixed to be 0.3 dB. It can be seen from the second column of the table that the loss in the subarray increases along with the increase in element number. The loss of a 2 × 2-element subarray is only about 0.3 dB, but that of 32 × 32-element subarray has increased to 4.8 dB. However, the smaller subarrays require more subarrays and the separate feed network is more complex. From the last column we can see that the minimum total insertion losses are achieved when 4 × 4- or 8 × 8-element subarray is used; both are 2.4 dB. Considering the complexity and cost of the separate feed network, the 8 × 8-element subarray is employed finally. In this subarray, the spacing between two adjacent elements is chosen to be 0.8 *λ*_0_, corresponding to 0.75 *λ*_01_ and 0.85 *λ*_10_. The size is 144 mm × 144 mm. The simulated results of S parameters for such a subarray is also shown in Figure 6. As can be seen, the frequency bands that |S_11_| below −10 dB are 12.1−12.76 GHz for the low band and 13.94−14.52 GHz for the high band, a little wider than those of the element. The simulated radiation patterns of the 8 × 8-element subarray for the lower and higher frequencies are shown in Figure 7. The resulting gains at 12.5 GHz and 14.25 GHz are 23.7 dBi and 25 dBi, respectively, the corresponding aperture efficiencies are 52% and 54%, respectively. The isolations between two different polarizations are 13.4 dB and 18 dB.

## 4. Experimental Results

### 4.1. Fabrication of the 32 × 32-Element Array

**Figure 8:**
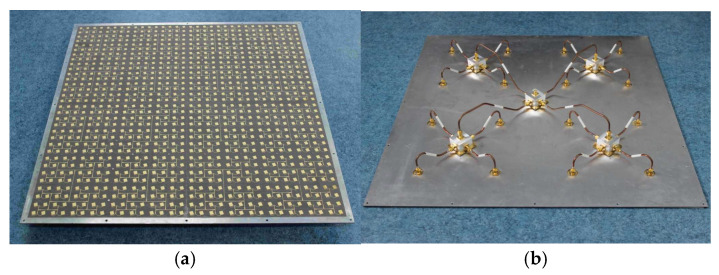
The fabricated 32 × 32-element antenna array. (**a**) Front view. (**b**) Back view.

The 8 × 8-element subarrays and low loss separate feed network are integrated together to compose a 32 × 32-element array. Figure 8 shows the photographs of the whole array. Sixteen 8 × 8-element subarrays are mounted uniformly on an aluminum plate in a 4 × 4 grid. The distance between two adjacent subarrays is 144 mm. The aperture size of the 32 × 32-element array is 576 mm × 576 mm. Each subarray is fed by a SMA connector in the center of the substrate bottom. The separate low-loss feed network on the other side of the aluminum plate is used to combine all the subarrays to one input port as shown in Figure 8b. It is constructed by 5 radial power dividers, 4 long semi-ridged coaxial lines and 16 short semi-ridged coaxial lines. All these components are connected by SMA connectors.

From Figure 8, we can see that the power from the input port is transformed by a long semi-ridged coaxial line, a short semi-ridged coaxial line and two radial power dividers, so the total loss in the separate feed network is the sum of the four components. To evaluate the insertion losses of different components, these components were measured using a Keysight vector network analyzer (Santa Rosa, CA, USA). Typical measured results are given in Table 2, where the losses caused by the SMA connectors have been included in the results. The insertion loss in the long semi-ridged coaxial line is about 0.1 dB higher than that in the short semi-ridged coaxial lines. The losses and phase differences between these lines are within 0.05 dB and 10 degrees, respectively. Due to their good performances, the influences caused by the difference between the lines can be ignored.

### 4.2. Bandwidth

**Figure 9:**
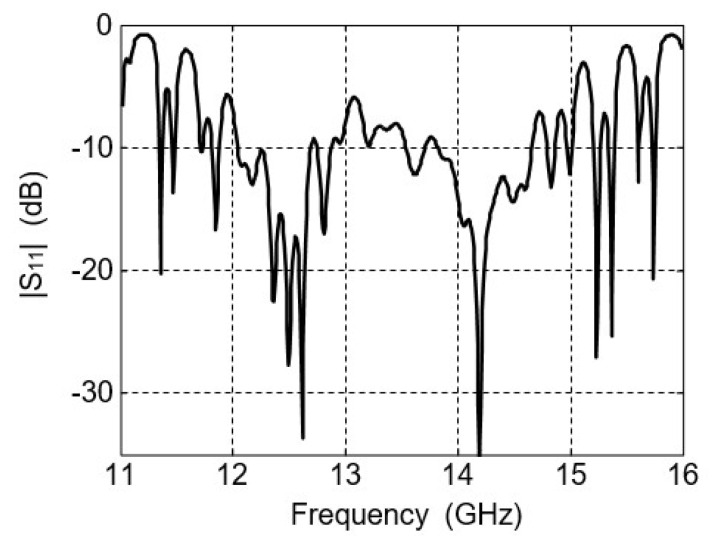
The measured |S_11_| of the 32 × 32-element antenna array.

The measured S parameter of the antenna array is shown in Figure 9. As can be seen, the frequency bands that |S11| below −10 dB for the two bands are 12.04−12.69 GHz and 13.82–14.66 GHz, respectively.

### 4.3. Radiation Patterns

**Figure 10:**
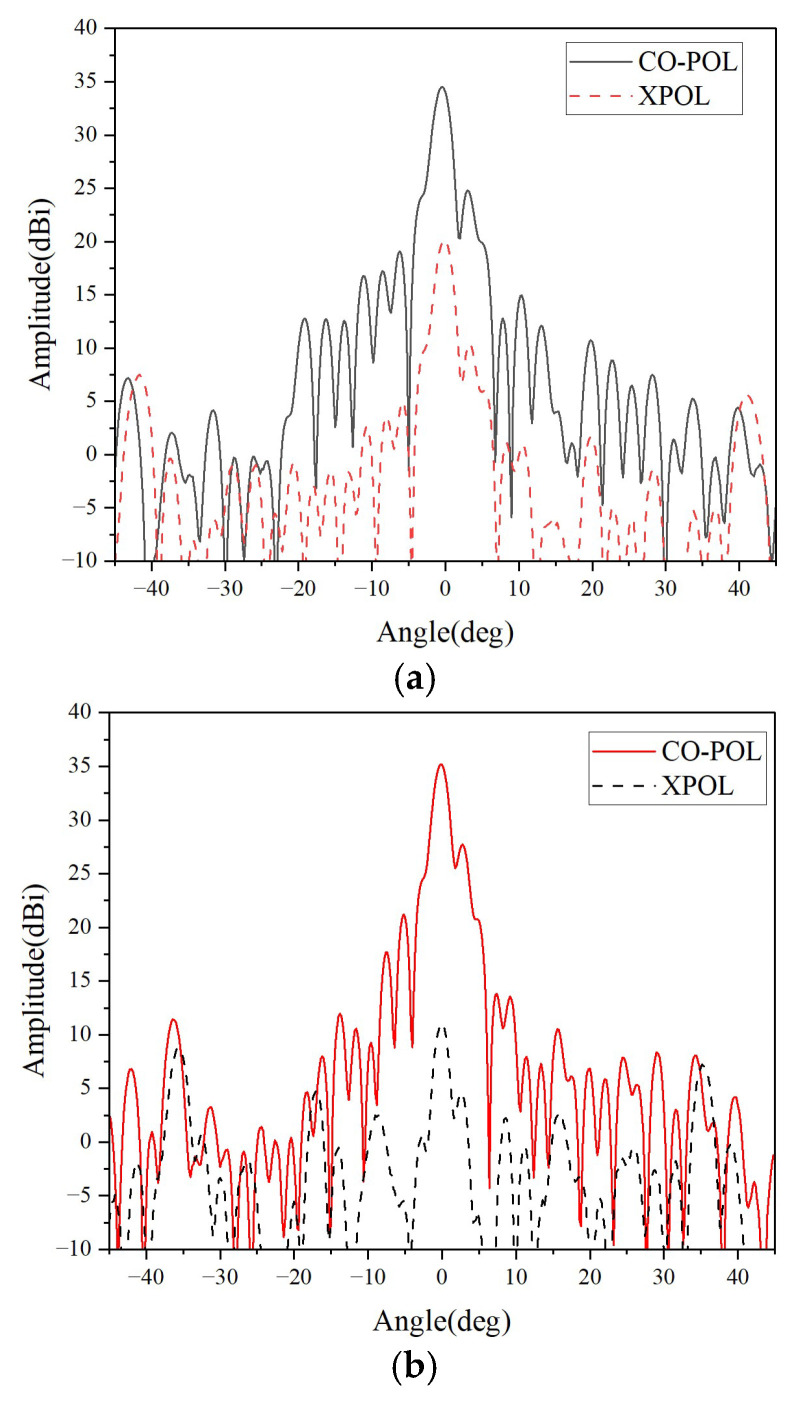
The Measured Radiation patterns (**a**) 12.5 GHz (**b**) 14.25 GHz.

To comprehensively evaluate the performance of this dual-frequency and dual-polarization antenna, we conducted a full set of measurements in a microwave anechoic chamber. The antenna radiation patterns measured at the center frequencies of 12.5 GHz and 14.25 GHz are shown in Figure 10.

In the low operating band (12.5 GHz), the antenna achieves a maximum gain of 34.5 dBi, with a corresponding aperture efficiency of 38.9%. The 3 dB main lobe width of the antenna is 2.2°, and the sidelobe level and cross-polarization level are −10 dB and −15 dB, respectively. In the high operating band (14.25 GHz), the antenna achieves a maximum gain of 35.2 dBi, with a corresponding aperture efficiency calculated as 35.2%. The 3 dB main lobe width of the antenna is 1.9°, while the sidelobe level and cross-polarization level are −8.5 dB and −25 dB, respectively. The measured results are basically consistent with the expected performance.

A comparison with prior dual-band dual-polarized antennas is shown in Table 3; most existing arrays are small-scale (2 × 2 or 8 × 8) with gains below 30 dBi. Our design overcomes this limitation via a low-loss power division network, achieving a gain of up to 35.2 dBi while maintaining low fabrication complexity.

## 5. Conclusions

A low-cost dual-frequency dual-polarization microstrip antenna array is designed and measured. The microstrip feed network is replaced by a low-loss separate feed network to reduce insertion losses. The dual-frequency dual-polarization operation is achieved by inserting a microstrip line into the corner of the rectangular patch. The measured results on a 32 × 32-element array show that the measured gains at the operating frequencies are 34.5 dBi and 35.2 dBi, respectively. This antenna array is suitable for satellite communication systems where compact size is a key requirement.

## Figures and Tables

**Table 1:** Design Parameters for the Power Divider.

Parameters	Initial Values	Final Values
*H*	*λ_g_*/2 = 15.32 mm	16.8 mm
*s*	*λ_g_*/4 = 7.66 mm	6.1 mm
*l*_1_	*λ_g_*/4 = 7.66 mm	6.0 mm
*l*_2_	*λ*_0_/2 = 5.61 mm	5.3 mm

**Table 2:** Loss Budgets of the Array.

Size of Subarray	Loss in Subarray (dB)	Loss in Separate Feed Network(dB)		Sum (dB)
		Power Divider Loss (dB)	Semi-Ridged Coaxial Lines Loss (dB)	
2 × 2	0.3	4 × 0.3 = 1.2	4 × 0.3 = 1.2	2.7
4 × 4	0.6	3 × 0.3 = 0.9	3 × 0.3 = 0.9	2.4
8 × 8	1.2	2 × 0.3 = 0.6	2 × 0.3 = 0.6	2.4
16 × 16	2.4	1 × 0.3 = 0.3	1 × 0.3 = 0.3	3.0
32 × 32	4.8	0	0	4.8

**Table 3:** Comparison with Prior Dual-Band Dual-Polarized Antennas.

Ref.	Gain dBi	Operating Band GHz	Sidelobe dB	Polarization Isolation dB	Aperture Efficiency	Fabrication Complexity
[6]	32.3	58.0–63.4 (8.8%)	−12	−43	60%	High
[10]	20.6 22.8	20 30	−10 −13.5	−25 −20	27% 23%	Medium
[15]	5.57 6.82	10.38 (1.1%) 13.9 (3.8%)	--	−13 −19	12.3% 11.1%	Medium
[16]	10.7 11.7	1.92–1.98 (9.2%) 2.11–2.17 (5.58%)	--	−21 −19	74.8% 78.2%	Low
[17]	4.6 3.3	1.42 1.79	--	−12 −10	--	Low
[19]	8.0 11.5	2.7 (7.4%) 8.1 (8.7%)	--	−18 −22	31.6% 7.9%	High
This work	34.5 35.2	12.04–12.69 (5.2%) 13.82–14.66 (5.9%)	−10 −8.5	−15 −25	38.9% 35.2%	Low

## Data Availability

The original contributions presented in this study are included in the article. Further inquiries can be directed to the corresponding author.
